# Cytotoxicity of Alizarine versus Tetrabromocathecol Cyclometalated Pt(II) Theranostic Agents: A Combined Experimental and Computational Investigation

**DOI:** 10.1021/acs.inorgchem.2c00842

**Published:** 2022-04-25

**Authors:** Gloria Mazzone, Stefano Scoditti, Rossella Caligiuri, Loredana Ricciardi, Emilia Sicilia, Maria Giovanna Lupo, Isabella Rimoldi, Nicolas Godbert, Massimo La Deda, Andreea Ionescu, Mauro Ghedini, Iolinda Aiello, Giorgio Facchetti

**Affiliations:** † Dipartimento di Chimica e Tecnologie Chimiche, Università della Calabria, Arcavacata di Rende, Cosenza 87036, Italy; ‡ MAT-InLAB, LASCAMM CR-INSTM, Unità INSTM della Calabria, Dipartimento di Chimica e Tecnologie Chimiche, Università della Calabria, Arcavacata di Rende, Cosenza 87036, Italy; § CNR NANOTEC, Institute of Nanotechnology U.O.S. Cosenza, Arcavacata di Rende, Cosenza 87036, Italy; ∥ Dipartimento di Medicina, Università degli Studi di Padova, Padova 35128, Italy; ⊥ Dipartimento di Scienze Farmaceutiche, Università degli Studi di Milano, Via Venezian 21, Milan 20133, Italy

## Abstract

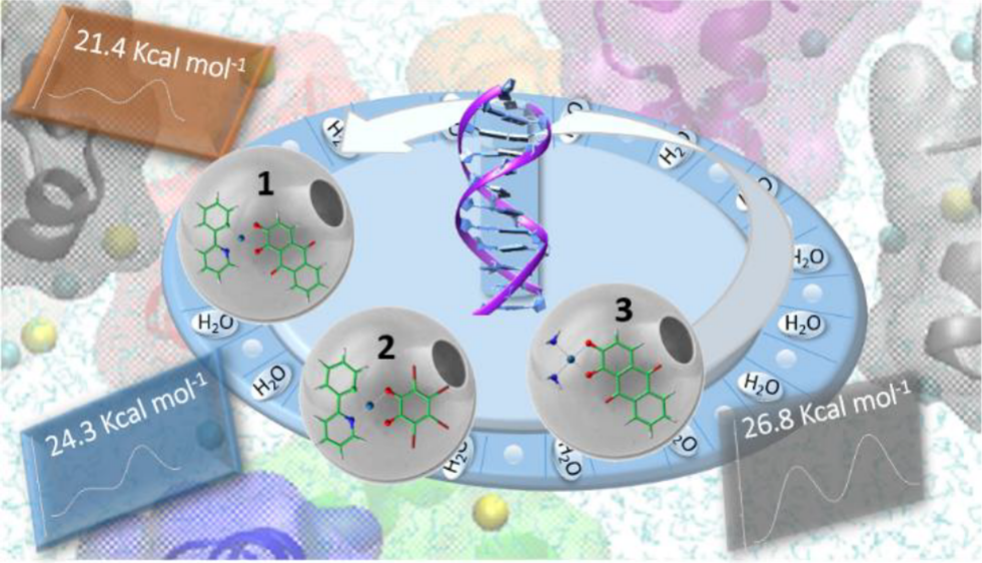

Platinum compounds cytotoxicity is strictly related to their ability to be converted into active mono- and di-aquated species and consequently to the replacement of labile ligands by water molecules. This activation process makes the platinum center prone to nucleophilic substitution by DNA purines. In the present work, quantum mechanical density functional theory (DFT) computations and experimental investigations were carried out in order to shed light on the relationship between the internalization, aquation, and DNA binding of two isostructural anionic theranostic complexes previously reported by our group, NBu_4_[(PhPy)Pt(Aliz)], **1 (**IC_50_ 1.9 ± 1.6 μM), and NBu_4_[(PhPy)Pt(BrCat)], **2 (**IC_50_ 52.8 ± 3.9 μM). Cisplatin and a neutral compound [(NH_3_)_2_Pt(Aliz)], **3**, were also taken as reference compounds. The computed energy barriers and the endergonicity of the hydrolysis reactions showed that the aquation rates are comparable for **1** and **2**, with a slightly higher reactivity of **1**. The second hydrolysis process was proved to be the rate-determining step for both **1** and **2**, unlike for compound **3**. The nucleophilic attack by the N7 site of guanine to both mono- and di-aquated forms of the complexes was computationally investigated as well, allowing to rationalize the observed different cytotoxicity. Computational results were supported by photostability data and biological assays, demonstrating DNA as the main target for compound **1**.

## Abstract 2

An experimental−theoretical approach to study the cytotoxicity of ionic Pt(II) complexes containing (O^O) chelating ancillary ligands.

## Introduction

Since their approval in medical protocols, cisplatin and its derivatives continue to play a central role as first-line therapy drugs for the treatment of different types of tumors.^1^ More than 40 years after the recognition of the cisplatin clinical benefits, studies still continue to be carried out in an effort to comprehensively understand its mechanism of action. Four main steps, internalization, aquation, formation of DNA adducts, and cell response, have been identified to explain the cytotoxic activity of cisplatin and its derivatives.^2,3^ Because the first step consists in drug internalization, its accumulation inside the cells surely represents the limiting step of its bioactivity. The cellular uptake of platinum drugs through the cellular membranes has been assumed to occur by both passive diffusion and through specific channels. In the last decades, compelling evidence for a more prominent role of carrier-mediated platinum drug uptake has rapidly accumulated in the literature.^4^ Inside the cells, the second step of the mechanism of action is aquation, which allows the activation of platinum drugs, thanks to the replacement of relatively labile ligands prone to nucleophilic substitution, such as chloride in cisplatin. The successive step of the mechanism of action involves the interaction between the active aquated complex with DNA. The cytotoxic activity of cisplatin is ascribed to its interaction with nucleophilic sites of purine bases in DNA, mainly the N7-site of guanine, to form inter- and intrastrand crosslinks^5^ inducing structural distortion. Following DNA damage, cells might repair themselves and then continue through the cell cycle, but if they are not able to repair the damage, cells will proceed to apoptosis.^6−8^ As widely demonstrated, the main species responsible for the DNA binding, in cisplatin, is the mono-aquated complex, *cis*[Pt-Cl(NH_3_)_2_(H_2_O)]^+^, while the reaction of the di-aquated species, which seems to be kinetically controlled, gives only a minor contribution to the binding between DNA and the platinum drug.^6,7^ It is worth mentioning, however, that some studies describe the di-aquated form of the drug as the major DNA-binding species because of the double charge and the better H-bond-donating capacity.^9^ With the aim of improving the cisplatin properties by the kinetic control of the aquation step, different complexes with ligands less prone to be displaced by water were synthesized such as carboplatin and oxaliplatin. Both of them differ from cisplatin in that they contain dicarboxylate chelate-leaving ligands.^10^ Such bidentate ligands, when hydrolysis takes place, are displaced in a stepwise fashion by the water nucleophile. In the case of carboplatin, the first hydrolysis causing the opening of the malonate ring is a very slow process compared to chloride substitution in cisplatin and also oxaliplatin, very likely due to the steric hindrance of the ligand.^11^ As a result, a monodentate malonate ligand is obtained, and the kinetic behavior depends on the acid conditions influencing the degree of protonation of the Pt(II) complex. The rate of labile ligand substitution reaction in Pt(II) complexes depends not only on the identity of the leaving ligands but also on the nature of the diamine ligands *trans* to them.^12^ On the basis of this assumption, in this work, we evaluated the relationship that should exist between the capability of internalization, aquation, and DNA binding, and the cytotoxic profile of two anionic Pt(II) complexes, previously synthetized by our research group,^13^ bearing two different chelate-leaving ligands, alizarine or tetrabromocatechol. Both these ligands allow the formation of ionic Pt(II) complexes and possess the same chelating O^O fragment, derived from 1,2-dihydroxybenzene. While alizarine possesses an extended aromatic system, the electronegative bromine atoms placed onto the catecholate ligand insures localization of the withdrawing electronic effect onto the chelated 1,2-dihydroxybenzene ring. Noteworthily, the cytotoxic activity of the two complexes is highly different. The alizarine derivative showed high response against human triple-negative breast cancer cell line MDA-MB-231, while the catecholate parent is rather inactive.^13^ To explain such disparity in behavior, a joint computational and experimental investigation was carried out. Quantum mechanical density functional theory (DFT) computations, supported by the detection of photophysical properties of the selected complexes, were employed to examine aquation steps, guanine binding, and deactivation interactions with model sulfur-containing molecules.

## Methods

### Computational Details

All the calculations were performed using DFT with Gaussian16 package.^14^ Solvent effects were included with an implicit model, the integral equation formalism variant of the polarizable continuum model (IEFPCM),^15^ using a dielectric constant of 78.4 to mimic the water environment. The hybrid B3LYP exchange and correlation functional,^16,17^ composed of Grimme’s dispersion correction (D3),^18^ was used for the optimization calculations in conjunction with the Pople double-ζ basis set 6-31+G* for the oxygen atoms and 6-31G** for C, N, and H atoms, while the effective core potentials (ecp) SDD^19^ and LANL2DZ^20,21^ were used for Pt and Br atoms, respectively, together with the split valence basis set. Local minima and transition states were identified by the number of imaginary vibrational frequencies (0 or 1, respectively). These calculations have been also used for obtaining Gibbs free energies at 298 K and 1 atm from total energies, including zero-point, thermal, and solvent corrections.^22^ Intrinsic reaction coordinate analysis was used to carefully check that each transition state is properly connected to the correct minima.^23,24^

Final energies were obtained by means of single-point calculations employing the triple-ζ basis set 6-311 + G** for all the atoms, except Pt and Br, for which the ecp def2-QZVP was employed in conjunction with the split valence basis set.^25,26^

The natural bond order (NBO) analysis^27,28^ has been performed by using Gaussian16 software at the same level of theory used for the optimizations.

UV–vis spectra were achieved, within the nonequilibrium time-dependent (TD) DFT approach,^29^ as vertical electronic excitations on the ground-state structure, using the same protocol used for the optimization calculations.

The theoretical estimation of the Log*P*_ow_ parameter for the complexes under investigation was obtained using the SwissADME tool, which calculates adsorption, distribution, metabolism, and excretion (ADME) parameters, pharmacokinetics, bioavailability, and drug-like behavior.^30^

### Reagents

High-glucose Dulbecco’s modified Eagle medium (HG-DMEM), trypsin–EDTA 0.05%, l-glutamine 200 mM, 10,000 U penicillin/10 mg mL^–1^ streptomycin solution, plates, and Petri dishes were supplied by EuroClone, and fetal bovine serum (FBS) was provided by Sigma-Aldrich. The compounds were dissolved in dimethyl sulfoxide (DMSO, Sigma-Aldrich) as 80 mM stock solutions, freshly prepared each time. The DMSO percentage tested by the cells did not exceed 0.25% v/v.

### Absorption Spectra

Spectrofluorimetric-grade solvents were used for the photophysical investigations. The buffer solution (pH 7.4) was prepared by dissolving one phosphate buffer saline table in 200 mL of water. Compounds **1**–**3** were dissolved in DMSO and then diluted to reach a final concentration of 1 × 10^–5^ M in DMSO as well as DMSO/buffer 0.5% v/v. A PerkinElmer Lambda 900 spectrophotometer was employed to obtain the UV–visible absorption spectra, using 10 mm path-length quartz cuvettes.

### Cell Culture

Human triple-negative breast cancer cell line, MDA-MB-231, was maintained in HG-DMEM supplemented with 10% v/v FBS, 1% v/v l-glutamine, and penicillin/streptomycin and subcultured at the ratio 1:4 upon reaching 90% of confluence. The cells were incubated in a humidified atmosphere at 37 °C and 5% CO_2_.

### Evaluation of DNA-Bound Pt(II)

First, 200,000 cells/well were seeded in the complete medium in a 12-well tray. The day after, the old media were discarded, and the cells were challenged with compound-containing media for 6 h (CisPt: 30 and 60 μM; **1**: 25 and 50 μM; **2**: 1 and 2 μM; **3**: 50 and 100 μM). An equal percentage of DMSO was added to control samples. The cells were thus washed two times with cold phosphate buffered saline (PBS) and digested with 700 μL of a homemade digest buffer (Tris 50 mM pH 8, NaCl 100 mM, EDTA 100 mM, and SDS 1%). The suspensions were moved to vials, and 400 μL of NaCl-saturated solution was added to them. The suspensions were thus vortexed and centrifuged at 12,000 rpm for 5 min. The supernatants were retrieved, added with 500 μL of 2-propanol, vortexed, and centrifuged at 12,000 rpm for 15 min to let DNA precipitate. DNA pellets were resuspended in 1 mL of ethanol 70%, vortexed, and centrifuged at 12,000 for 4 min. DNA pellets have been resuspended in 100 μL of homemade TE buffer (Tris 10 mM pH 8, EDTA 0.1 mM) and incubated at 37 °C for 10 min. DNA-bound Pt(II) was quantified through inductively coupled plasma-mass spectrometry (ICP-MS), and data were normalized to DNA content quantified with NanoDrop Site.

### Evaluation of the Intracellular Pt(II) Content

First, 200,000 cells/well were seeded in complete medium in a 12-well tray. The day after, the old media were discarded, and the cells treated with compound-containing media for 6 h at the following concentrations: cisplatin: 30 and 60 μM, **1**: 25 and 50 μM, **2**: 1 and 2 μM, **3**: 50 and 100 μM. These concentrations correspond to IC_50_ and IC_50_/2 of each compound according to our previous work aimed at analyzing the cytotoxic performance of these Pt(II) complexes in the MDA.MB.231 cell line.^13^ Accordingly, the cisplatin IC_50_ on the same cell line was reported by our group in a previous study.^31^ An equal percentage of DMSO was added to control samples. Cells were thus washed two times with cold PBS and lysed with 200 μL of a homemade lysis buffer (Tris 50 mM pH 7, NaCl 150 mM, NP40 1% v/v, protease, and phosphatase inhibitors 1% v/v) for 30 min at 4 °C. The intracellular Pt(II) amount was quantified through ICP-MS (BRUKER aurora M90 ICP-MS, MA, USA), and data were normalized to the total protein content evaluated via bicinchoninic acid assay.

### DNA Binding Experiment: UV–vis Absorption Evaluation

In a quartz cuvette (path length 1 cm), the UV–vis spectra were recorded of a PBS buffer solution (pH 7.4)-containing complex (**1**, **2**, or **3**, 50 μM, 0.5% DMF). The UV–vis absorption spectra were successively recorded following each addition of aliquots of Calf Thymus DNA (CT-DNA, 50 μM) after an equilibration time of 1 h.^32,33^

### DNA-Binding Experiment: Fluorescence DNA Competitive Study

To a solution in PBS buffer containing the system CT-DNA (10 μM) with ethidium bromide (EtBr, 5 μM), increasing amounts of complex (**1**, **2**, or **3**, 5 μM, in 1% v/v DMF) were added, and the displacement by the complex of EtBr from the system CT-DNA/EtBr was evaluated by measuring the decrease in the intensity of the recorded emission.^32,33^

### Experimental Measurement of Log*P*_ow_

Reverse phase high performance liquid chromatography (RP-HPLC) analysis was performed to correlate the hydrophobicity of the complexes with their retention time. The chromatograms were analyzed using reversed-phase HPLC column (Partisil C18-ODS), at 25 °C, using KI as the internal standard and as the mobile phase water/methanol in ratio 80/20 in the presence of 15 mM HCOOH (flow rate: 1 mL/min, λ = 210 nm). The calibration curve was realized in comparison with reference compounds (cisplatin, carboplatin, and oxaliplatin).^31,34^

## Results and Discussion

**Chart 1:**
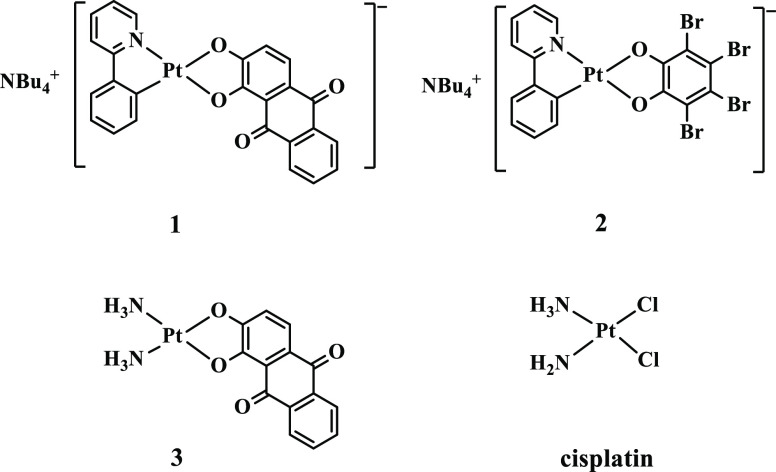
Chemical Structure of Cyclometalated Anionic Pt(II) Complexes **1**–**3** and Cisplatin

Part of the research activities carried out by some of us has been recently focused on the synthesis and properties of anionic organometallic complexes.^35−37^ In particular, the theranostic activity of two series of anionic Pt(II) complexes of general formula NBu_4_[(C^N)Pt(O^O)], characterized by the presence of a cyclometalated (C^N) ligand (2-phenylpyridine, H(PhPy), 2-thienylpyridine, H(ThPy), or 2-benzo[*h*]quinoline, H(Bzq)), and by two different (O^O) chelate ligands, alizarine H_2_(Aliz) or tetrabromocatechol H_2_(BrCat), was reported previously.^13^ Noteworthy, in our previous study,^13^ the complex named **1,** bearing (PhPy)^−^ and (Aliz)^2–^ ligands, showed a significant cytotoxic activity with IC_50_ 1.9 ± 1.6 μM against human triple-negative breast cancer cell line MDA-MB-231. Complex **1** cytotoxic activity is considerably higher than that of the other synthesized complexes, especially its analogue **2** in which (BrCat)^2–^ is the (O^O) chelate ligand (IC_50_ 52.8 ± 3.9 μM) and complex named **3**, where the (C^N) ligand is substituted by two ammonia molecules (IC_50_ 126.9 ± 2.7 μM) (Chart 1).^13^ Such significant differences in terms of cytotoxicity, considering that **1** and **2** possess identical (C^N) ligands, point out that the (O^O) chelated ligand must play a key role as the leaving group and, accordingly, it was herein investigated in depth. On the other hand, complex **3** was considered because of the parent of the cisplatin archetype, and it was taken as a reference compound along with cisplatin. The comparison between complex **1** and **3** will allow to probe the effect of the (C^N) ligand with respect to two ammonia ligands onto the aquation processes.

## Aquation Reaction, Guanine Binding, and Interaction with *N*-Acetyl Methionine

**Figure 1:**
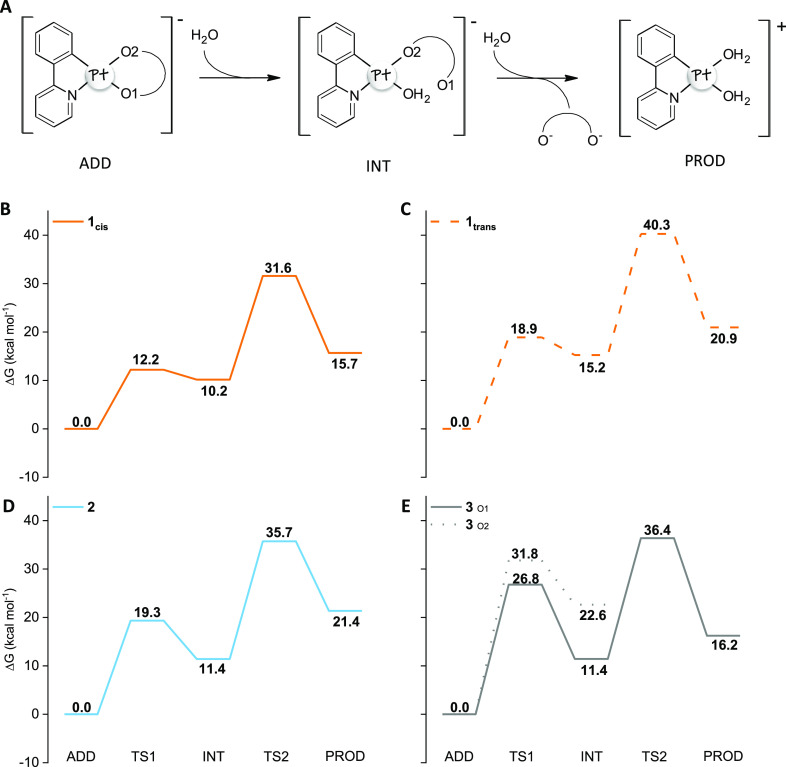
(A) Hydrolysis reaction scheme. Free energy profiles for the aquation reaction in water solvent of (B) **1_cis_** (solid orange line), (C) **1_trans_** (dashed orange line), (D) **2** (light blue line), and (E) **3** (solid gray line) causing the O1 detachment. For complex **3** also the O2 detachment (dotted gray line) is depicted. ADD, starting adduct; TS1, transition state for the first hydrolysis; INT, mono-aquo Pt(II) complex; TS2, transition state for the second hydrolysis; PROD, di-aquo Pt(II) complex.

**Figure 2:**
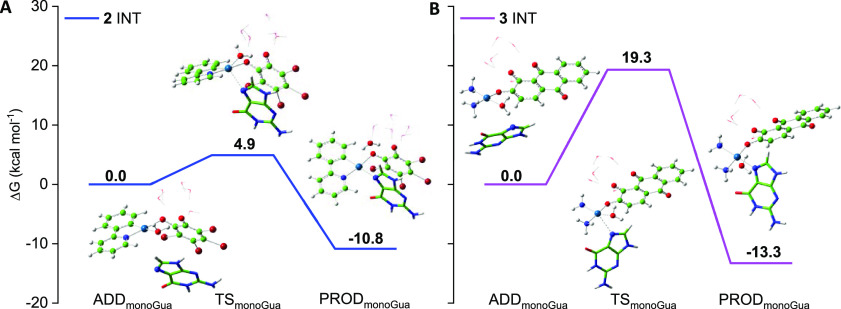
Free energy profiles describing the guanine attack in the water solvent for the displacement of water to monoaquoated forms, INT, of (A) anionic **2** (blue line) and (B) neutral **3** (purple line) complexes.

Aiming at understanding which is the origin of the difference in cytotoxic profiles of the aforementioned complexes (Chart 1), computational studies were first exploited in order to explore the key steps of the mechanism of action of Pt(II) anticancer drugs, that is, ligand substitution by water, leading to the formation of the corresponding aqua-complexes and the subsequent binding to nuclear DNA, whose structure is thus distorted. Calculations were carried out for anionic complexes **1** and **2** and for the neutral one **3**. The calculated energy profiles illustrating both aquation and guanine-modeled DNA platination are shown in Figures 1 and 2, respectively. The optimized structures of the intercepted stationary points for aquation steps are reported in Figure S1 of the Supporting Information, while those for guanine binding are also included in Figure 2.

As widely reported throughout in the literature, the hydrolysis reaction of square-planar d^8^ complexes is realized according to an associative mechanism in which the displacement of the leaving group by a water molecule passes through the formation of a pseudo-trigonal bipyramid transition state,^38^ following the scheme included in Figure 1, drawn for the two anionic complexes **1** and **2**. As explained in our previous studies,^38−40^ to take into consideration the explicit solvent environment, in the starting adduct (ADD), the Pt(II) complexes were surrounded by six solvent molecules, which establish a network of hydrogen bonds with the oxygen atoms of the (O^O) leaving group.

The first hydrolysis product, INT, is obtained with the detachment of an oxygen atom of both (BrCat)^2–^ or (Aliz)^2–^ ligands through the five-coordinated transition state TS1. The final di-aquo complex, PROD, is formed by the definitive release of the (O^O) ligand and the concerted water molecule entrance in the first coordination shell of the Pt(II) center overcoming the energy barrier corresponding to a penta-coordinated transition state (TS2). Because of the asymmetric structure of the (C^N) PhPy ligand, the water attack and the detachment of the oxygen atom of the leaving ligand can occur in the *trans* or in *cis* position with respect to the C-coordinated atom of PhPy. Preliminary calculations performed on **1** evidenced that because of the trans effect, such attack takes place preferentially in the *trans* position to the carbon atom of the PhPy ligand. Thus, only the mechanism for the displacement of the O1 atom by water is reported for the investigated anionic complexes.

In the case of complex **1**, given the asymmetry even of the (Aliz)^2–^ leaving ligand, two geometric isomers were taken into consideration, in which the quinone part of (Aliz)^2–^ lies on the same side, **1_cis_**, or on the opposite side, **1_trans_**, of the N-coordinated atom of the (C^N) ligand (see Figure S2 of the Supporting Information). The calculated comparable stabilities of the two isomers (Δ*E* = 0.6 kcal mol^–1^) fit with the ^1^H-NMR evidence of an approximately 1:1 cis/trans ratio.^12^ The first hydrolysis of **1_cis_** occurs overcoming a low-energy barrier of 12.1 kcal mol^–1^, while 18.9 kcal mol^–1^ is required for the aquation of **1_trans_** (Figure 1B,C, respectively). The reaction is endergonic by 10.2 and 15.2 kcal mol^–1^ for **1_cis_** and **1_trans_**, respectively. The second hydrolysis requires 21.4 and 25.0 kcal mol^–1^ to occur in **1_cis_** and **1_trans_**, respectively. In both cases, the whole hydrolysis reaction is endergonic by 15.7 kcal mol^–1^ for the *cis* isomer and 20.9 kcal mol^–1^ for the *trans* one. From these data, it emerges that both isomers can undergo the (O^O) ligand substitution by two water molecules, even if both hydrolysis steps are easier to occur on the *cis* than the *trans* isomer, from both kinetic and thermodynamic points of view. Indeed, as the positions of the two oxygen atoms are interchanged in the two isomers, the different charge distribution, due to the asymmetric nature of the alizarine ligand, is interchanged as well. The oxygen atom on the side of the quinone moiety, that is, the O1 atom in the *cis* isomer, is less nucleophilic, as shown by the NBO analysis^27,28^ and, then, more prone to be displaced by water. Analogous to the cisplatin-leading compound,^41,42^ the second hydrolysis of **1**, in both isomers, represents the rate-determining step of the whole process. Compared to cisplatin for which the first and second hydrolysis reactions are reported to be endergonic (by 3.6 and 4.2 kcal mol^–1^, respectively) and the estimated energy barriers range from 19 to 23.3 kcal mol^–1^,^41,43−45^ computations confirm the aquation viability for complex **1**.

Despite the different identity of the (O^O) ligand, a similar behavior was found for complex **2** (Figure 1D). Indeed, the second water substitution remains the slowest step of the whole reaction and occurs overcoming an energy barrier of 24.3 kcal mol^–1^, and both the first and second hydrolysis steps are endergonic of 11.4 and 21.4 kcal mol^–1^, respectively, likewise the energy expense required for the aquation reaction of **1_trans_**.

A remarkable difference, instead, was found for the neutral complex **3**. In this case, both being ancillary ligand ammonia molecules, no trans effect could be hypothesized, and both water attacks on the oxygen atoms named O1 and O2 were taken into consideration. Then, both first detachments of O1 or O2 allowing the entrance of a water molecule were considered equally viable. As it is evident in Figure 1E, the O1 detachment (solid gray line) is the most favored one from both kinetic and thermodynamic points of view, and the water attack from the side of O2 seems to be hampered because no stationary points describing the second hydrolysis step were located, despite the numerous attempts. The substantial difference with respect to the anionic complexes relies on the involved energy barrier for the first hydrolysis (26.8 kcal mol^–1^), that is higher than those computed for both **1** and **2**, and represents the rate-limiting step of the whole process in the case of **3**. The (Aliz)^2–^ ligand displacement by water definitively takes place by overcoming an energy barrier of 25.0 kcal mol^–1^, and the reaction product (PROD) lies 16.2 kcal mol^–1^ above the zero reference energy of the initial adduct (ADD).

Therefore, looking at the energy barriers computed for the complexes under examination as well as the endergonicity of the whole hydrolysis reaction, it clearly appears that the aquation rates are comparable to each other with only a slightly higher reactivity of **1_cis_**.

Aquation reaction is propaedeutic to the subsequent DNA platination step in the mechanism of action of anticancer Pt(II) complexes achieved through the attack of the aquated complex to DNA nucleobases. Computational studies highlighted that the N7 site of the guanine nucleobase represents the preferred site of attack by platinum-based drugs, leading to DNA distortion.^46^ Thus, the guanine attack to both mono-aquo and di-aquo complexes was investigated. It is worthy of note that the di-aquo complex obtained from both (BrCat)^2–^ or (Aliz)^2–^ displacement is the same, [(PhPy)Pt(H_2_O)_2_]^+^, while that coming from complex **3** di-aquation is the same as that obtained from cisplatin hydrolysis, [(NH_3_)_2_Pt(H_2_O)_2_]^2+^. Nevertheless, for the sake of comparison, also the latter case was taken into consideration.

Guanine attack to displace water from the mono-aquated form of complex **3** was examined only for the intermediate that was calculated to be more kinetically accessible. The results of this exploration are reported in Figure 2 for both **2** and **3** complexes, whereas all the efforts carried out to locate the stationary points along the corresponding path for complex **1** were unsuccessful. As it is evident in Figure 2, the substitution reaction in the mono-aquated complex **2** is very favorable from both kinetic and thermodynamic points of view. Indeed, only 4.9 kcal mol^–1^ is required to overcome the activation energy barrier, and the reaction is exergonic by 10.8 kcal mol^–1^.

On the contrary, the height of the energy barrier is 19.3 kcal mol^–1^ when the attack of guanine to the mono-aquated form of complex **3** is taken into consideration, whereas the thermodynamics continues to be favorable, the reaction being exergonic by 13.3 kcal mol^–1^. This computationally evidenced difference in reactivity of mono-aquo complexes might be one of the factors useful to rationalize the observed different toxicities.

**Figure 3:**
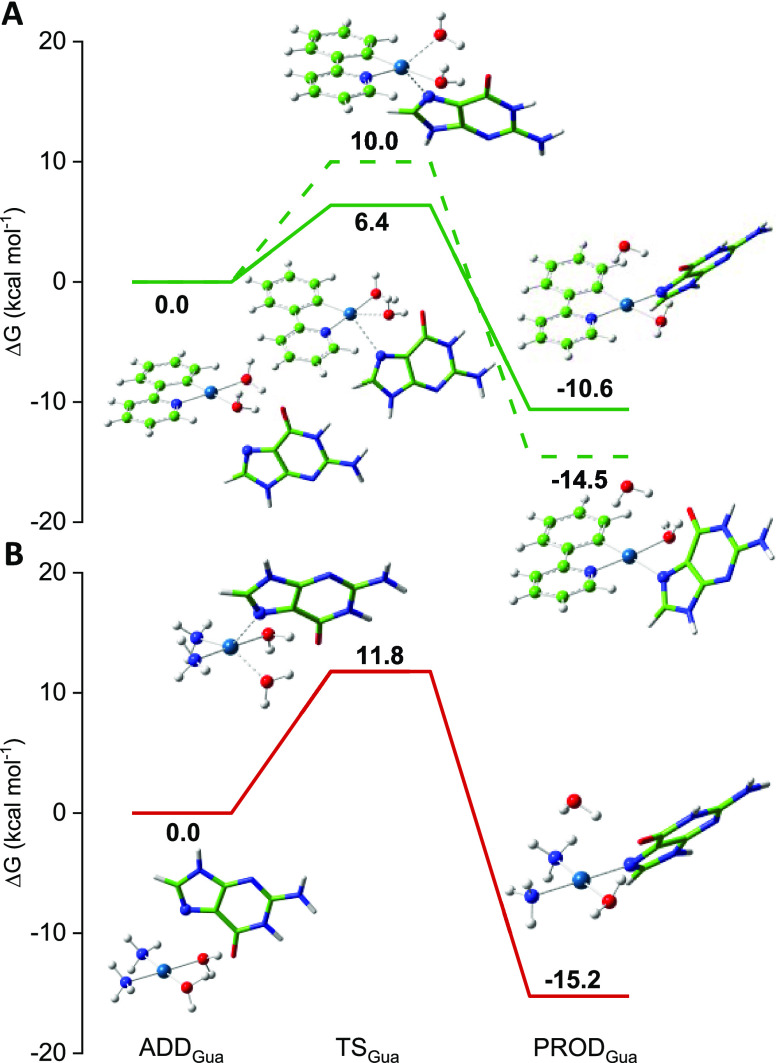
Free energy profiles describing the guanine attack in the water solvent to (A) di-aquo complex [(PhPy)Pt(H_2_O)_2_]^+^ for the displacement of water in the cis (solid green line) or in the trans position (dashed green line) with respect to the N-coordinated atom and (B) di-aquo complex [(NH_3_)_2_Pt(H_2_O)_2_]^2+^.

Upon examining the water displacement by guanine from the di-aquo complex, what reported above for the aquation mechanism was taken into consideration, and given the asymmetry of the PhPy ligand, the attack on both *cis* and *trans* positions with respect to the C-coordinated atom of the (C^N) ligand was investigated. Water detachment from the platinum center and guanine binding to it occur, once again, following a S_N_2 associative mechanism going through a penta-coordinated transition state TS_Gua_. The first interaction adduct ADD_Gua_ is characterized by a guanine orientation that can be preparatory to both attacks. However, the attack of guanine from the same side of the N-coordinated atom requires only 6.4 kcal mol^–1^ to take place (solid green line in Figure 3A), and it is energetically favored with respect to the attack from the other side (10.0 kcal mol^–1^, dashed green line in Figure 3A). The displacement reaction is exergonic by 10.6 kcal mol^–1^ in the former case and by 14.5 kcal mol^–1^ in the latter. On the other hand, guanine displacement by water in the [(NH_3_)_2_Pt(H_2_O)_2_]^2+^ complex occurs overcoming an energy barrier higher than those just discussed (11.8 kcal mol^–1^), whereas the product PROD_Gua_ is stabilized by 15.2 kcal mol^–1^ with respect to the reference adduct.

**Figure 4:**
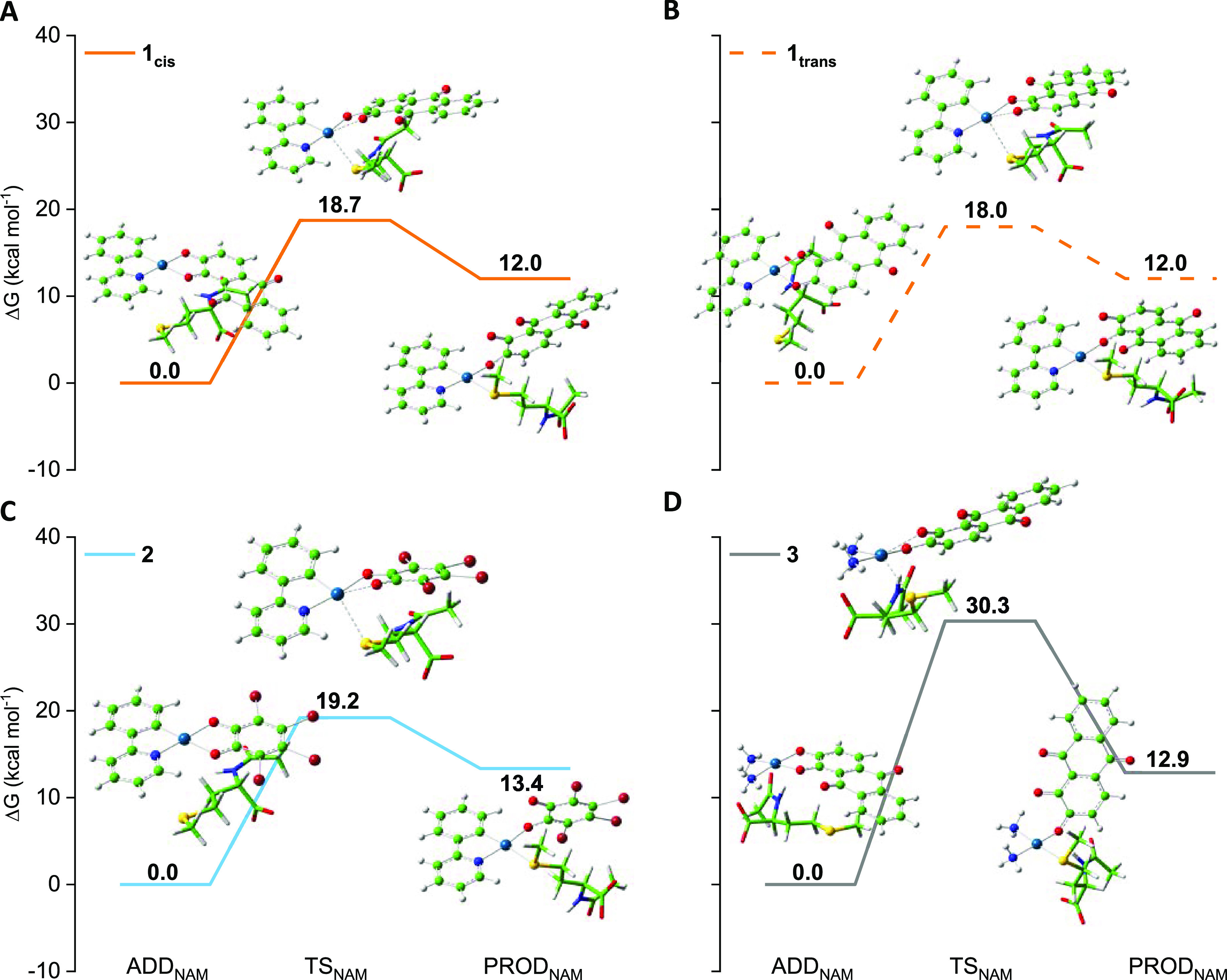
Free energy profiles describing the NAM attack to (A) **1_cis_** (solid orange line), (B) **1_trans_** (dashed orange line), (C) **2** (light blue line), and (D) **3** (gray line).

Upon searching for a difference in behavior justifying the observed different cytotoxicity, given the soft nature of platinum, the possibility that the complexes can be deactivated by the interaction with sulfur-containing biological molecules was taken into account. *N*-acetyl methionine (NAM) was used as a model to explore the tendency of the investigated complexes to undergo the attack of sulfur-containing biological residues, and the resultant free energy profiles, describing the interaction of **1**, **2**, and **3** with NAM, are provided in Figure 4.

On the basis of the outcomes of the investigations reported above, showing that the trans position to the C-coordinated atom of the (C^N) ligand is the preferred attack of both water and guanine molecules, the same site was taken into consideration for the substitution reaction of an oxygen atom of the (O^O) ligand by NAM. As usual, the reaction starts with the initial interaction of the platinum complex and the approaching NAM ligand forming the first adduct (ADD_NAM_) and proceeds through a pseudo-trigonal bipyramidal transition state (TS_NAM_). In the formed product (PROD_NAM_), the (O^O) ligand remains mono-coordinated to the metal center, and NAM occupies the coordination site in trans to the C-coordinated atom. Even in this case, the anionic complex **1**, for both *cis* and *trans* isomers, and **2** do not show substantial differences. The attack of NAM requires 18.7 and 18.0 kcal mol^–1^ to take place in **1_cis_** and **1_trans_**, respectively, compared to an energy barrier of 19.2 kcal mol^–1^ computed in the case of **2**. In addition, the detachment of one arm of the (O^O) ligand in favor of NAM coordination is calculated to be endergonic in all cases, being 12 kcal mol^–1^ for both **1** isomers and 13.4 kcal mol^–1^ for **2**. Also, in the case of the neutral complex **3,** formation of the NAM substitution product causes a similar destabilization by 12.9 kcal mol^–1^ even if, from a kinetic point of view, the substitution reaction is hard to occur (Δ*G*^‡^ = 30.3 kcal mol^–1^). Table 1 summarizes the energies involved in the activation steps that are aquation and guanine binding, and a plausible deactivation process here explored, that is the interaction with a model sulfur-containing residue like NAM.

**Table 1:** Calculated Activation (Δ*G*^‡^) and Reaction (Δ*G*_rxn_) Free Energies (kcal mol^–1^) in Water Describing the First and Second Aquation Processes, Guanine, and NAM Interactions

	1st aquation		2nd aquation		guanine interaction				NAM interaction	
					mono-aquo		di-aquo			
	Δ*G*^‡^	Δ*G*_rxn_	Δ*G*^‡^	Δ*G*_rxn_	Δ*G*^‡^	Δ*G*_rxn_	Δ*G*^‡^	Δ*G*_rxn_	Δ*G*^‡^	Δ*G*_rxn_
**1_cis_**	12.2	10.2	21.4	15.7			6.4	–10.6	18.7	12.0
**1_trans_**	18.9	15.2	25.0	20.9			“	“	18.0	12.0
**2**	19.3	11.4	24.3	21.4	4.9	–10.8	“	“	19.2	13.4
**3**	26.8	11.4	25.0	16.2	19.3	–13.3	11.8	–15.2	30.3	12.9

At a first glance, from the data reported in Table 1, it appears that the *cis* isomer of complex **1** is more prone to aquation and the difference is more evident for the first aquation reaction. On the other side, the slowest step results to be the second hydrolysis for both complexes **2** and **1**, in its *cis* and *trans* conformations (21.4 and 25.0 kcal mol^–1^ for **1_cis_** and **1_trans_**, respectively, vs 24.3 kcal mol^–1^ for **2**). The high activation energy calculated for the hydrolysis (26.8 kcal mol^–1^) of complex **3** could explain the very high IC_50_ measured for such complex.^13^ Guanine binding is easily accessible, as expected, for all di-aquo complexes even if a difference in the barrier height exists between the aquated form of **1** and **2** complexes and that of complex **3**. Furthemore, from the reported data, it emerges that, in addition to a similar propensity of **1** and **2** to undergo aquation and, then, guanine attack, even a possible deactivation by the attack of sulfur-containing species seems equally probable.

## Optical Properties

The photophysical properties of **1**–**3** in DMSO have been previously discussed.^13,35^ In order to investigate the tendency of the Pt(II) complexes to undergo hydrolysis reactions giving the corresponding aqua-complexes, their stability in aqueous solution was monitored spectrophotometrically, acquiring the absorption profiles over time (*t* = 0, 3, 6, and 24 h). In particular, absorption spectra were registered by dissolving **1**–**3** in DMSO then diluted in buffer solution (DMSO 0.5% v/v). Moreover, in order to follow a possible aquation reaction with a slower kinetics, absorption spectra were recorded dissolving **1–3** in DMSO containing a low water content, by using a nonanhydrous spectroscopic-grade solvent.

**Figure 5:**
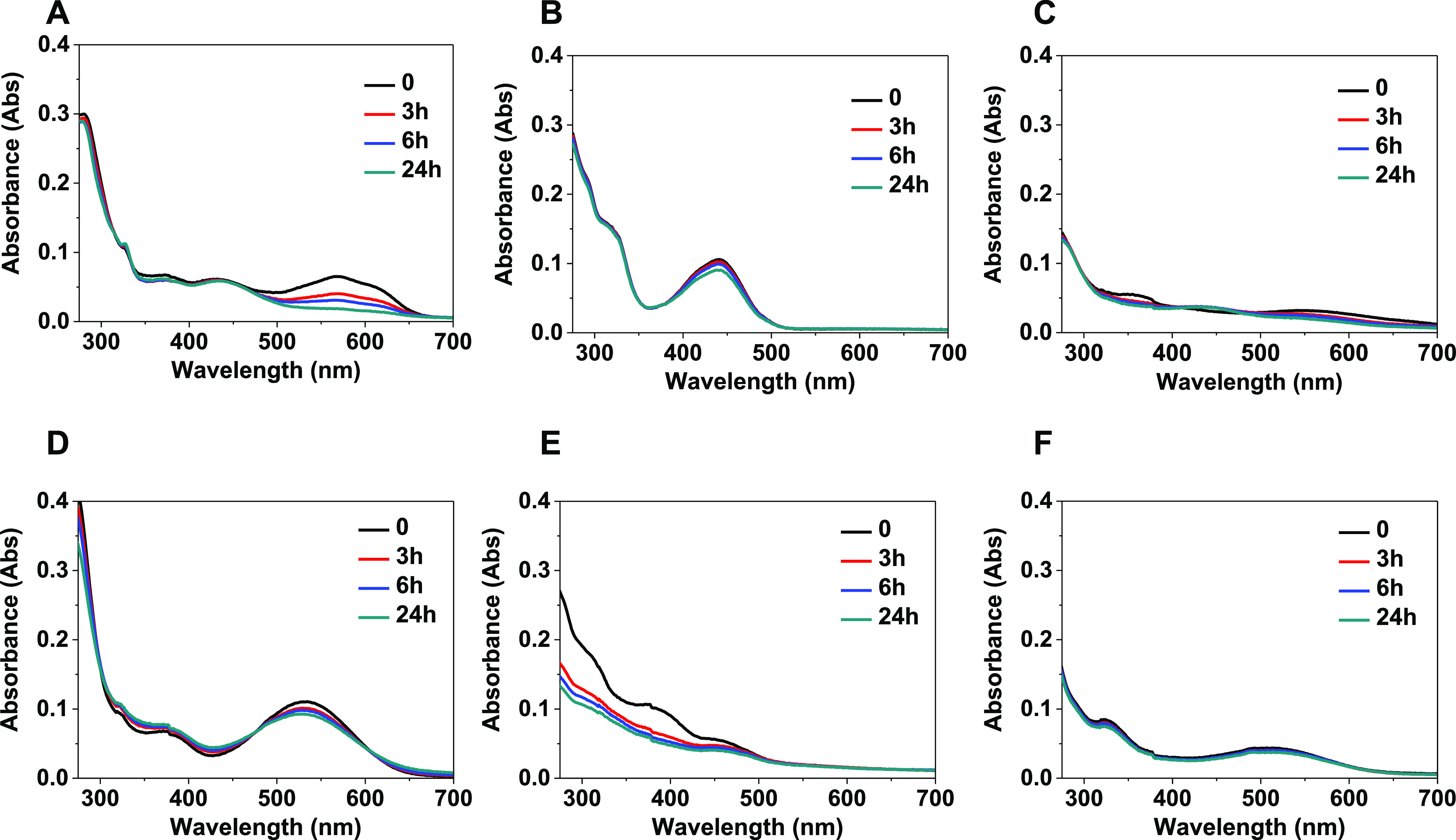
Absorption spectra over time (*t* = 0, 3, 6, and 24 h) of Pt(II) complexes (A) **1**, (B) **2**, and (C) **3** in DMSO and of (D) **1**, (E) **2**, and (F) **3** in buffer solution containing 0.5% v/v DMSO.

Figure 5A shows the absorption spectra in DMSO of the anionic compound bearing the (Aliz)^2–^ ligand. While in the higher energy regions of the UV–visible spectrum, no significant profile changes were observed, low-energy absorption bands between 600 and 650 nm—attributed to metal–ligand charge-transfer (MLCT) transitions, predominantly involving the alizarine fragment^13^—gradually decrease over time, pointing out a possible displacement of the chelate ligand. In DMSO/buffer solution (Figure 5D), the absorption spectrum of **1** is markedly blue-shifted with respect to the spectrum recorded in DMSO and does not show noteworthy changes in the 0–24 h time range, plausibly suggesting a rapid and immediate aquation reaction. Furthermore, the slight increase of the 375 nm band suggests that this spectral feature may be typical of the aquated species.

The electronic spectra in DMSO and in DMSO/buffer solution of the anionic compound bearing the (BrCat)^2–^ ligand are shown in Figure 5B, E, respectively. In particular, the absorption spectrum of **2** in the DMSO solution appears stable over time, with a slight decrease, mostly evident after 24 h, of the broad low-energy band between 380 and 600 nm. The latter was attributed to an MLCT transition involving the catecholate fragment,^35^ indicative of, as in the previous case, a possible detachment of the chelate ligand but with a slower kinetics with respect to that observed for the anionic compound bearing the alizarine leaving group. The spectral profile of **2** in aqueous medium (Figure 5E) shows evident changes compared to that in DMSO solution. While the ligand-centered (LC) transitions localized on the cyclometalated ligand (between 280 and 350 nm) appear unchanged, a new pronounced peak at 379 nm and a considerable decrease of the charge-transfer band were observed. These features suggest that in aqueous solution, the displacement of the catecholate ligand occurs, whereas the new band could be due to the mono or di-aquated compound. By observing the absorption spectrum of **2** in aqueous solution over time, a reduction in the optical density was observed for the whole spectral range, plausibly due to a poor solubility of the compound in the aqueous medium.

Finally, the absorption spectra of **3** are shown in Figure 5C, F. At *t* = 0, the absorption spectrum of the neutral compound in DMSO (Figure 5C) displays a weak peak at 357 nm and a broad band centered at 555 nm ascribed, as for the relative compound **1,** to a charge-transfer transition from the metal center to the alizarine ligand. After 3 and then 6 and 24 h, the spectral shape changes, showing a progressive decrease of the band at 357 nm as well as at 555 nm, whereas a shoulder at 440 nm becomes gradually much more pronounced. Also in this case, the decrease in the MLCT transition suggests a potential detachment of the leaving ligand, whereas the other spectral variations could be due to the simultaneous presence in solution of **3**, its aquated form, and the free alizarine. Going from the DMSO to the DMSO/buffer solution (Figure 5F), the absorption spectra do not show any change over time, except for a slight decrease in the optical density probably resulting from the poor solubility of the neutral compound in aqueous solution. Therefore, in order to confirm the spectral attributions and shed light on the spectral features of the mono and di-aquated species, time-dependent density functional theory (TD-DFT) calculations were performed.

The absorption spectra in implicit DMSO and water solvents of the complexes and all their plausible derivatives that can be formed by aquation reaction were computed including the mono- and di-aquo complexes and the free ligand. The outcomes of these calculations are reported in Figures S3, S4, and Table S1, where the absorption spectra of all the species, the natural transition orbitals computed for the most important transitions in water, and detailed information on each transition are reported. These data confirm that, as experimentally observed, the solvent environment considerably influences the relative intensity of the absorption bands. In the high-energy region of the spectrum (400–650 nm), the band is due to an MLCT transition in all cases. In the spectra of both isomers of complex **1**, the intensity of such band, generated by HOMO → LUMO electronic transition, decreases with the detachment of an oxygen atom of the (O^O) ligand in the mono-aquo complex and completely disappears for the di-aquo complex in both solvents. In the aqueous environment, for the mono-aquo complex of **1_cis_** and **1_trans_**, such a band is blue-shifted by 57 and 24 nm, respectively. Because the recorded spectra within 24 h did not exhibit such behavior, it is reasonable that the **1** complex is rapidly converted into the di-aquo species. Therefore, the decrease in such band intensity in the explored time (Figure 5A, D) is compatible with the complete release of the alizarine ligand, and the persistence of such a band even after the (O^O) ligand detachment can be ascribable to the presence of the free ligand in solution; indeed, looking at Figure S3, it appears clear that the intensity of such a band, that becomes the π → π* type, decreases with respect to the MLCT peak found for both intact and mono-aquo complexes. The computed spectra and the relative NTOs evidence that the high-energy band is originated even by a MLCT transition involving the PhPy ligand that falls in the same region of the LC band.

A different behavior was observed for complex **2**. The MLCT in the low-energy region involves the PhPy ligand, and it is originated by the electronic transition HOMO → LUMO+1. In contrast to complex **1**, in the case of **2**, when the mono-aquo complex is formed, its spectral features do not involve such region of the spectrum, thus the band around 450 nm completely disappears. This would suggest that most of the **2** complex remains intact in the investigated temporal range. However, the shape of the mono-aquo complex also differs in the region 280–350 nm, which in this case is characterized by an additional peak. Such a peak was experimentally observed as well (379 nm), indicating that only the first aquation should occur. Nevertheless, differently from the free (Aliz)^2–^ ligand, the spectrum of (BrCat)^2–^ falls in the shorter wavelength region and is characterized by decreased band intensities (see Figure S3).

Analogously, in the case of the neutral complex **3**, the appearance of the shoulder at 440 nm can be ascribed to the formation of the mono-aquo complex. In such cases, an interesting feature was observed. Comparing the spectrum of **3** with that of the free ligand, different from complex **1**, the intensity of the band in the region 400–600 nm is more for the free ligand than that for the complex, thus the decrease of such a band experimentally found is incompatible with the complete release of the (O^O) ligand. Hence, the persistence in the intensity of the LC band and the slight decrease of the MLCT band suggest that the alizarine remains anchored to the metal center, being di- or at least mono-coordinated.

Therefore, photophysical features support the hypothesis that in the aqueous environment, while complex **1** undergoes water attack leading to the complete detachment of the (O^O) ligand, most probably, both **2** and **3** complexes within 24 h are converted to the corresponding mono-aquo complexes.

## Pharmacological Evaluation

**Figure 6:**
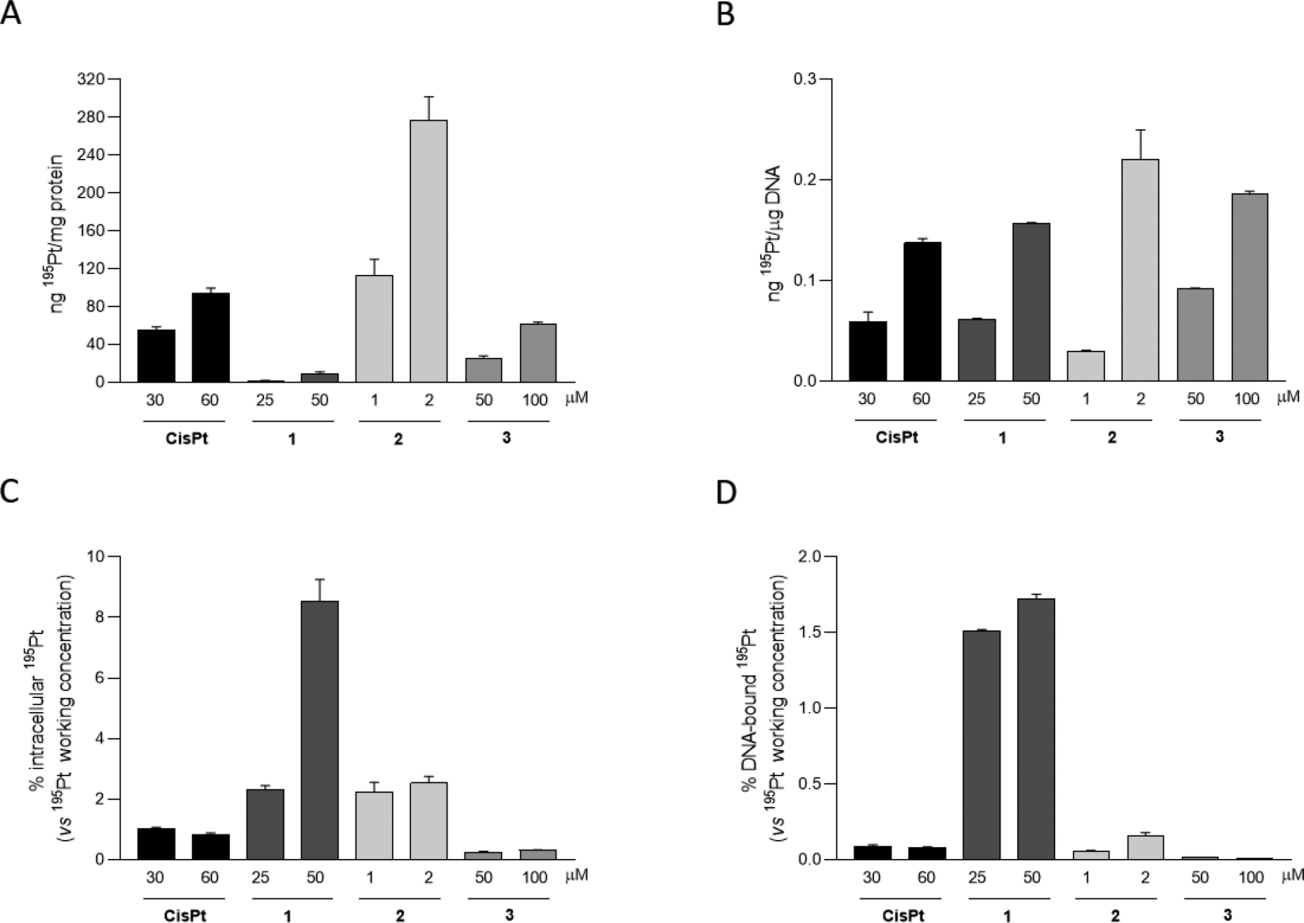
Intracellular and DNA-bound ^195^Pt in MDA-MB – 231 cell line upon 6 h of incubation with Pt(II) complexes and cisplatin. (A and C) Cells were seeded in complete medium (400,000 cells/well); the day after old media were discarded and cells were incubated for 6 h with media-containing compounds at the indicated concentrations, then intracellular protein were extracted, and intracellular ^195^Pt was evaluated as ng ^195^Pt over protein concentration (A) and further normalized vs the compounds’ working concentrations provided (C). Cisplatin was used as the positive control. An equal amount of DMSO was supplied in each experimental condition. Data are presented as mean ± SD of three different experiments; when invisible, the error bar is included into the bar line. (B and D) Cells were seeded in complete medium (200,000 cells/well); the day after old media were discarded and cells were incubated for 6 h with media-containing compound at the indicated concentrations, then DNA was extracted, and DNA-bound ^195^Pt was evaluated as ng ^195^Pt over DNA concentration (B) and further normalized vs the compounds’ working concentrations provided (D). Cisplatin (CisPt) was used as the positive control. An equal amount of DMSO was supplied in each experimental condition. Data are presented as mean ± SD of three different experiments; when invisible, the error bar is included into the bar line. CisPt: cisplatin.

With the aim to rationalize the difference in the cytotoxic profiles of the investigated Pt(II) complexes,^13^ an evaluation of the Pt(II) compound subcellular compartmentalization was realized. Although the studied concentrations of the different compounds are different in values, because of the disparities in IC_50_ and IC_50_/2 of each compound, these concentrations induced cytotoxicity to a similar extent among the different complexes. However, in order to take into account this difference, we normalized the obtained results (ng Pt/mg total protein and ng Pt/μg DNA) vs the concentration of each compound incubated to the cells. Thus, by presenting the data as % of Pt bound to protein and DNA, we eliminated the bias of the different utilized concentrations utilized. Complex **3** was studied as the reference compound considering that, as already highlighted above, it is formed by the coordination of platinum with two ammonia as main amine ligands and with (Aliz)^2–^ as the leaving group. The cells were incubated with **1**, **2**, **3**, and cisplatin for 6 h at the indicated concentrations, and then, the intracellular Pt(II) content and the Pt(II) bound to DNA were evaluated via ICP-MS (Figure 6).

Interestingly, while **2** at the highest tested concentration was very effective in binding to intracellular proteins compared to the highest cisplatin dose (3-fold, Figure 6A), at the same concentration, we observed a modest increase (1.6-fold, Figure 6B) in DNA-bound ^195^Pt compared to cisplatin 60 μM, suggesting a more efficient entrance in the cells compared to cisplatin and a slight preference toward the cytosolic compartment rather than the nucleus. These observations were also confirmed upon correction vs the **2** working concentration provided to the cells (Figure 6C, D). Conversely, **1** barely localizes into cytosol, compared to cisplatin and the other compounds (Figure 6A), while it seems to prefer the nucleic environment. Indeed, as shown in Figure 6B, its behavior is pretty superimposable to cisplatin one, except that **1** working concentrations were an order of magnitude lower than cisplatin, and this may explain its so low IC_50_ compared to cisplatin and to the other tested compounds.^13^**1** preferential subcompartimentalization was further highlighted upon correction for the initial working concentration provided. As shown in Figure 6D, we indeed observed a significant 10-fold higher accumulation of platinum in the DNA after treatment with 2 μM **1** compared to cisplatin 60 and 150 μM **2**, thus suggesting that DNA is its main cellular target compared to **2** and that its potent cytotoxic action could be due to a molecular mechanism similar to cisplatin. Moreover, as already underlined, the complex **1** possess the same cyclometalated amine (C^N) ligand of compound **2** but a different leaving group that probably plays an important role in the internalization of the compounds inside the cells causing the differences in terms of cytotoxicity of the two compounds.

## In Vitro DNA Binding Experiment

A practical method for assessing the binding affinity in vitro between a Pt(II) complex with DNA is the use of UV–vis and fluorescence spectroscopy employing Calf Thymus DNA (CT-DNA) mimicking the DNA structure and function also in association with ethidium bromide (EtBr), whose displacement with a Pt(II) complex can be ascribed to a non-negligible interaction.

Considering DNA results to be one of the main targets for the studied complexes, the interaction between CT-DNA and the complexes **1**, **2**, or **3** was evaluated by UV–vis spectroscopy. The spectra of the complex with an increasing amount of CT-DNA were recorded, and a decrease of the band at 545 nm, assigned to the π–π* transition (MLCT band) between the leaving group and the Pt(II) metal center, was observed as the competitive binding with the CT-DNA took place (see Figure S5). The same behavior was evidenced by fluorescence emission spectra. In the competitive study, the experiments were realized by adding different amounts of complex to the fluorescent system CT-DNA/EtBr. Upon the addition of complexes **1–3**, a dramatic decrease in the fluorescence intensity of the emission band of the CT-DNA/EtBr system was observed (see Figure S6). These results obtained for both experiments indicated a strong binding affinity between our complexes and CT-DNA, confirming the data obtained by computational and pharmacological evaluations.^32,33^

## Theoretical and Experimental Lipophilicity Evaluation

Lipophilicity is a pivotal drug property that impact drug uptake and metabolism. It is, indeed, the major determining factor in a compound absorption, distribution in the body, penetration across membranes and biological barriers, metabolism, and excretion (ADME properties). The Lipinski’s lipophilicity rule affirmed that one of the parameters for drug-likeness is that the log*P*_ow_ should be lower than 5. Both calculated and experimentally determined values of log*P*_ow_ for the three complexes examined in the present study are reported in Table 2 and compared with the measured values for the three FDA-approved Pt(II) drugs cisplatin, carboplatin, and oxaliplatin,^47^ and the calculated value for alizarine alone. Theoretical and experimental values, in very satisfactory agreement, have been determined to be positive for all the three complexes, although log*P*_ow_ for complex **3** is significantly lower than those of **1** and **2** complexes. The value for complex **2** (∼4.5) in turn is larger than that of complex **1**. Assuming the cellular uptake of platinum drugs through the cellular membranes by passive diffusion to be strictly correlated to their lipophilicity, complex **1**, with a log*P*_ow_ around 3, shows a desirable balance between lipophilicity and hydrophilicity.

**Table 2:** Experimental and Theoretical log*P*_ow_ Values of Pt(II) Complexes and Alizarine

compound	log*P*_ow_^exp^	log*P*_ow_^theo^
alizarine	1.87	2.02
**1**	3.33	3.08
**2**	4.53	4.67
**3**	0.47	0.67
cisplatin	–2.53^a^	
carboplatin	-2.30^a^	
oxaliplatin	–1.76^a^	

a From ref (47).

It is worth underlining that the estimation of such a parameter is just a preliminary indication of more intricate aspects, such as uptake, efflux, cell distribution, and nuclear concentration that play a pivotal role in defining the cytotoxic profile of an anticancer drug. Computational studies that focused on modeling such features are still in progress and, without any doubts, they all influence the description of the whole mechanism of action.

## Conclusions

In order to gain some insights able to support the most significant cytotoxic effect, previously reported by our group, against MDA-MB-231 exerted by compound **1** (IC_50_ 1.9 ± 1.6 μM), if compared to **2** (IC_50_ 52.8 ± 3.9 μM) and to the relative negligible toxicity shown by **3** (IC_50_ 126.9 ± 2.7), the hydrolysis process to the corresponding active mono- and di-aquated forms was explored by DFT calculations. In the case of complex **1**, the asymmetric structure of the labile alizarine leaving group was taken into consideration, showing for **1_cis_** a lower energy barrier than **1_trans_** both in the first (12.1 kcal mol^–1^ vs 18.9 kcal mol^–1^) and in the second water substitution reactions (21.4 kcal mol^–1^ vs 25.0 kcal mol^–1^). For both **1** and **2**, the second hydrolysis reaction proved to be the rate-determining step, while for the reference compound **3**, a higher energy barrier (26.8 kcal mol^–1^) was computed for the formation of the mono-aquated species. Indeed, the absorbance of the complexes **1**–**3**, monitored spectrophotometrically within 24 h both in DMSO and in diluted phosphate buffer solution (DMSO 0.5% v/v, pH 7.4) and computationally reproduced, suggest that the di-aquo complex is most probably formed only in the case of complex **1**.

Starting from the assumption that DNA is the main target responsible for platinum chemotherapeutics’ cytotoxicity, the nucleophilic attack by the N7 site of guanine to displace water from the aquated active forms was evaluated as well. Taking into consideration the asymmetric structure of the PhPy ligand in the water displacement reaction by guanine from the di-aquated forms of **1** and **2**, the nucleophilic attack by N7 of guanine from the side containing the N-coordinated atom resulted energetically favored, following a S_N_2 associative mechanism through a penta-coordinated transition state TS_Gua_, and requiring only 6.4 kcal mol^–1^ to take place. The energy barrier calculated for compound **3**, indeed, proved to be higher when considering the attack by N7 of guanine on both the mono- and di-aquo species (19.3 and 11.8 kcal mol^–1^, respectively). This, in part, supports the different cytotoxicity exhibited by the selected Pt(II) complexes against the triple-negative MD-MBA-231 cancer cell line. In the attempt to comprehensively elucidate this aspect, a plausible deactivation process was taken into consideration by examining the reaction with a well-known deactivating sulfur-containing molecule, NAM. Finally, ICP-MS analysis was performed by incubating the MDA-MB-231 cancer cell line with compounds **1**, **2**, **3**, and cisplatin up to 6 h, revealing complex **1** preference for DNA with a 10-fold higher concentration of DNA-bound platinum at 2 μM if compared to the one afforded by cisplatin at 60 μM. The same behavior was evidenced also in comparison to the two complexes **2** and **3**. The data obtained with in vitro DNA-binding experiments corroborated the assumption that DNA is the main target of the herein-proposed complexes. The values of lipophilicity also confirmed that compound **1** possesses a right balance between lipophilicity and hydrophilicity (log*P*_ow_ about 3); on the contrary, the compound **2** probably has a too high value (log*P*_ow_ about 4.5). This experimental evidence supports the ICP-MS analysis that the different leaving group may play an important role in the internalization of the compounds inside the cells; after that, the differences in terms of aquation and guanine interaction processes are not so evident as testified by computational and experimental data. The synthesis of new Pt(II) complexes containing alizarine is currently under progress in our laboratories to further investigate the role of the ligands on their cytotoxic activity, in particular to investigate the difference brought by the introduction of different electronegative substituents onto the 2-phenylpyridine ligand.

## Supporting Information Available

Optimized structures of the stationary points intercepted along the hydrolysis potential energy surfaces of the investigated complexes. Computed absorption spectra in implicit water and DMSO solvents of the investigated Pt(II) complexes. Natural transition orbitals for MLCT and LC bands. Key information on electronic excitations computed in water and DMSO. UV–vis spectra of CT-DNA in the presence of investigated complexes. Emission spectra of CT-DNA with EtBr in the presence of investigated complexes (PDF)
