# Supplementary material for: Cytotoxicity of Alizarine versus Tetrabromocathecol Cyclometalated Pt(II) Theranostic Agents: A Combined Experimental and Computational Investigation
Source: Inorg Chem. 2022 Apr 25;61(18):7188–200. doi: 10.1021/acs.inorgchem.2c00842 (PMC9092348; doi:10.1021/acs.inorgchem.2c00842)
Supplement: Supplementary file 1 — ic2c00842_si_001.pdf [file ic2c00842_si_001.pdf]

Supplementary

Cytotoxicity of alizarine versus tetrabromocathecol cyclometalated Pt(II) theragnostic agents: a combined experimental and computational investigation

Gloria Mazzone[1], Stefano Scoditti[1], Rossella Caligiuri[2], Loredana Ricciardi[3], Emilia Sicilia[1], Maria Giovanna Lupo[4], Isabella Rimoldi*[5], Nicolas Godbert[2], Massimo La Deda[2,3], Andreea Ionescu[2], Mauro Ghedini[2], Iolinda Aiello*[2,3], Giorgio Facchetti[5]

[1]*Dipartimento di Chimica e Tecnologie Chimiche, Università della Calabria, 87036 Arcavacata di Rende (CS), Italy*

[2]*MAT-InLAB, LASCAMM CR-INSTM, Unità INSTM della Calabria, Dipartimento di Chimica e Tecnologie Chimiche, Università della Calabria, 87036 Arcavacata di Rende (CS), Italy*

[3]*CNR NANOTEC-Istituto di Nanotecnologia UOS Cosenza, 87036 Arcavacata di Rende (CS), Italy*

[4]*Dipartimento di Medicina, Università degli Studi di Padova, 35128 Padova, Italy*

[5]*Università degli Studi di Milano, Dipartimento di Scienze Farmaceutiche, Via Venezian 21, 20133 Milan, Italy*

Table of Contents

Figure S1: Optimized structures of the stationary points intercepted along the hydrolysis potential energy surfaces of **1**, as isomer A) *cis* (solid orange line) or B) *trans* (dashed orange line), of C) **2** (light blue line) and D) **3** for the O1 (solid grey line) or O2 detachment (dotted grey line) attacks **S2**

Figure S2: Optimized structures of the investigated Pt(II) complexes **S4**

Figure S3: Computed absorption spectra in implicit A) water and B) DMSO solvents of the investigated Pt(II) complexes and free ligands $(Aliz)^{2-}$ and $(BrCat)^{2-}$ **S5**

Figure S4: Natural Transition Orbitals for MLCT and LC bands **S6**

Table S1: Wavelength, λ (nm), oscillator strengths, *f*, and main transitions computed in water and DMSO **S7**

Figure S5: UV-Vis spectra of different concentration of CT-DNA in PBS buffer solution in the presence of complexes **1-3** (50 μM) **S8**

Figure S6: Emission spectra of CT-DNA (10 μM) with EtBr (5 μM) after the addition of increased amount of complexes **1-3**. **S9**

Figure S1. Optimized structures of the stationary points intercepted along the hydrolysis potential energy surfaces of **1**, as isomer A) *cis* (solid orange line) or B) *trans* (dashed orange line), of C) **2** (light blue line) and D) **3** for the O1 (solid grey line) or O2 detachment (dotted grey line) attacks

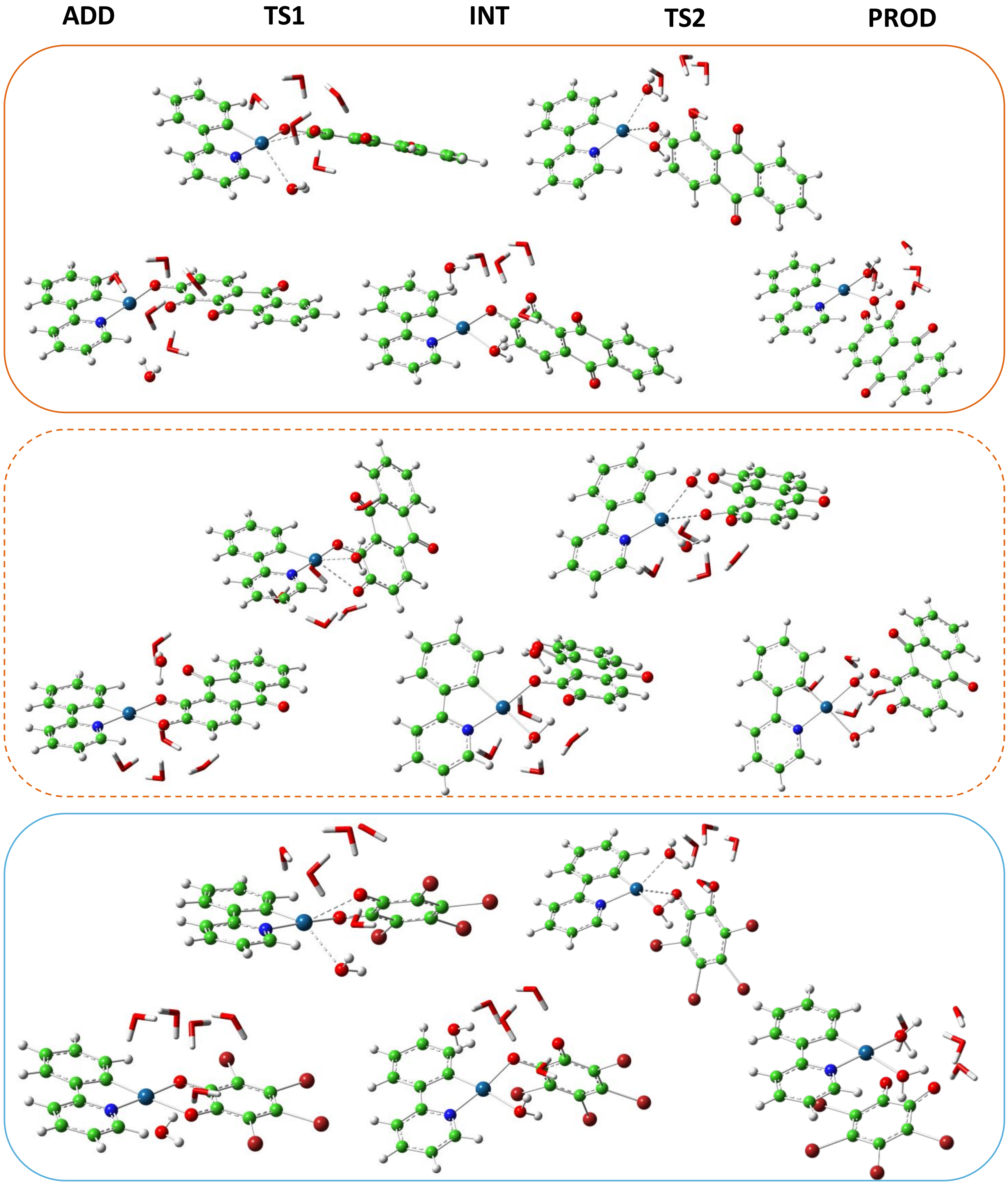

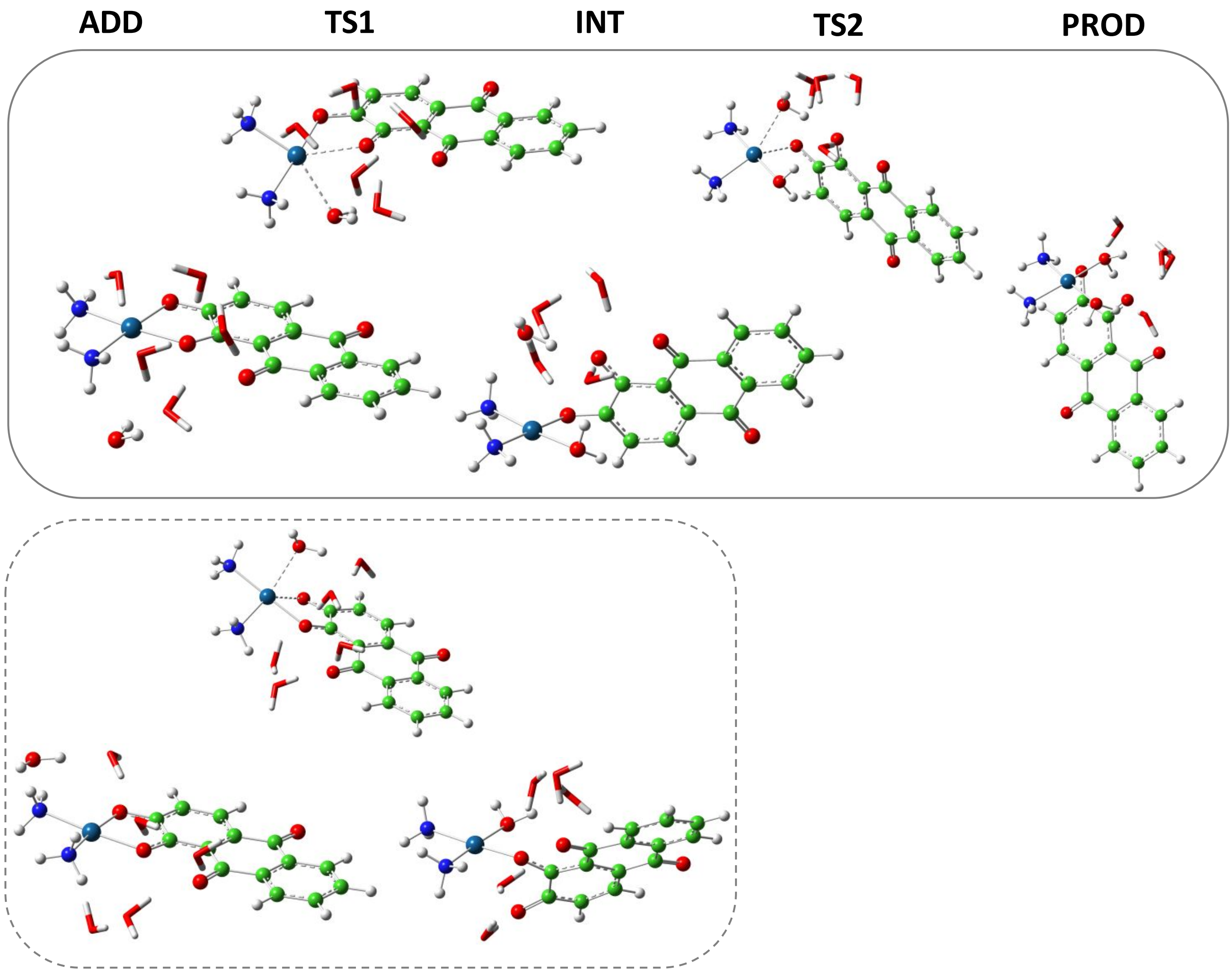
ADD
TS1
INT
TS2
PROD

Figure S2. Optimized structures of the investigated Pt(II) complexes

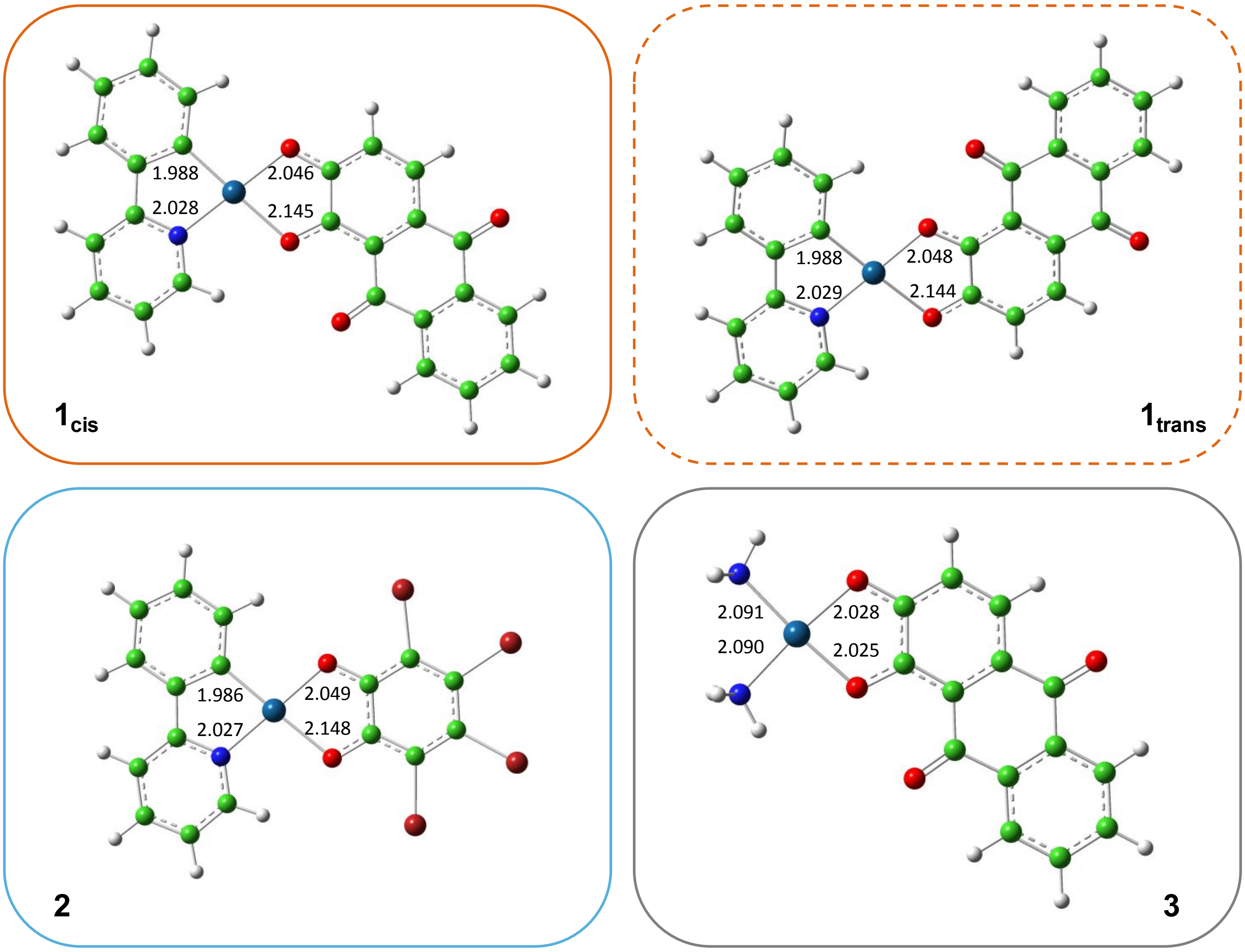

Figure S3: Computed absorption spectra in implicit A) water and B) DMSO solvents of the investigated Pt(II) complexes and free ligands $(Aliz)^{2-}$ and $(BrCat)^{2-}$.

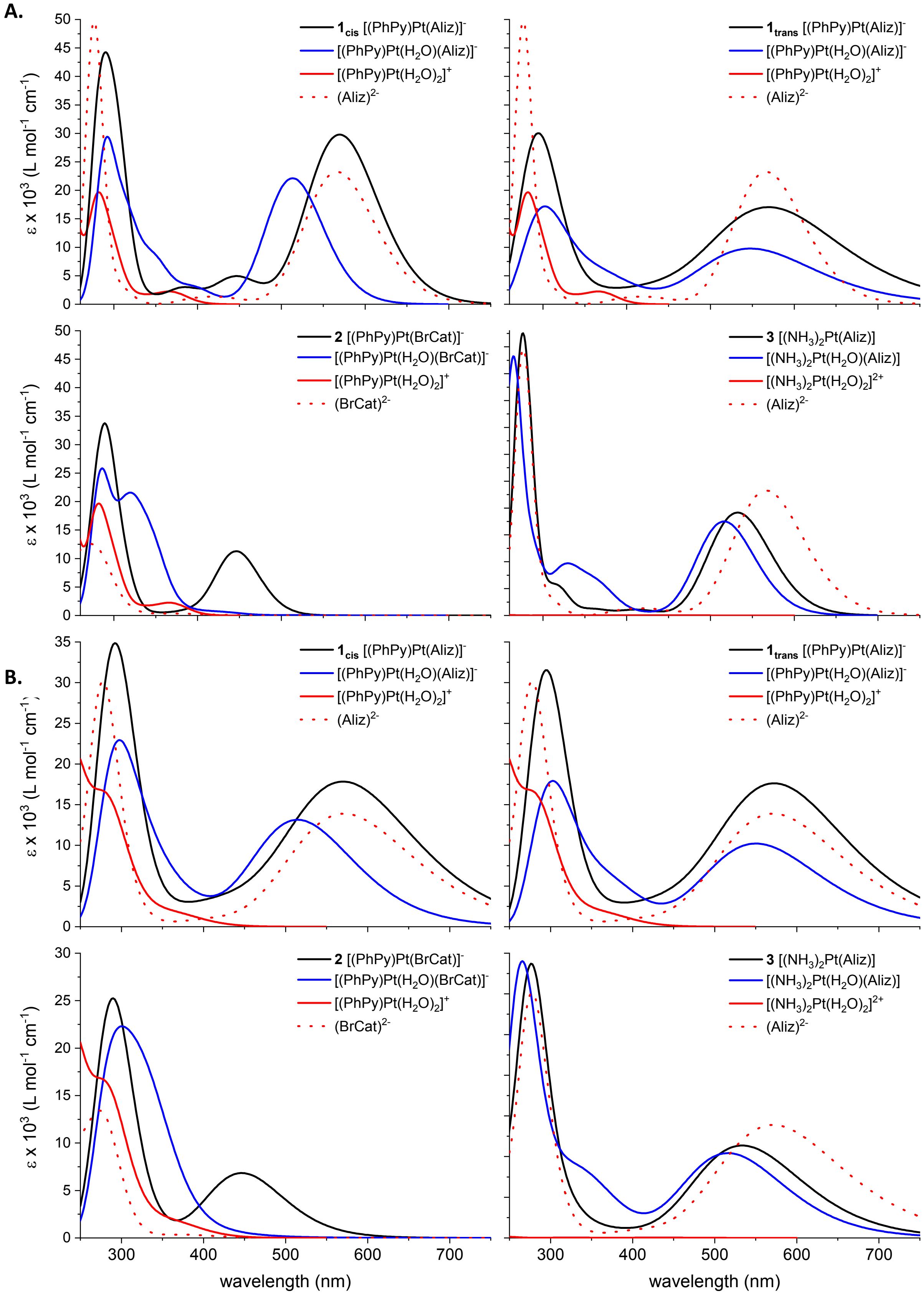

Figure S4: Natural Transition Orbitals for MLCT and LC bands

	400 – 600 nm *hole → particle* / 280 – 350 nm *hole → particle*
**1**$_{cis}$ $[(PhPy)Pt(Aliz)]^-$	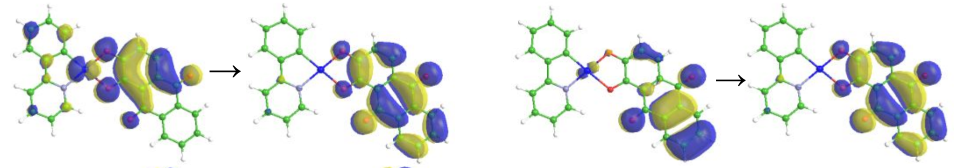
$[(PhPy)Pt(H_2O)(Aliz)]^-$	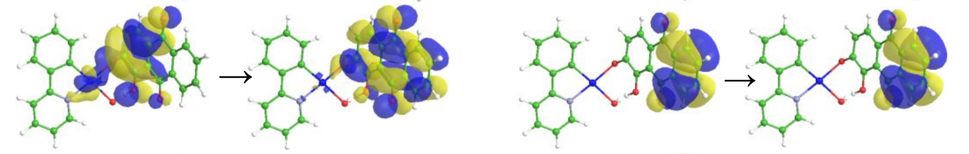
**1**$_{trans}$ $[(PhPy)Pt(Aliz)]^-$	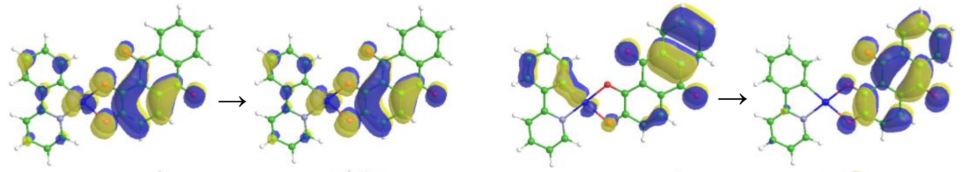
$[(PhPy)Pt(H_2O)(Aliz)]^-$	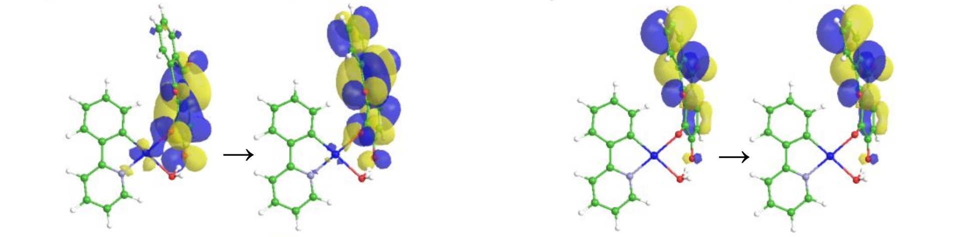
$[(PhPy)Pt(H_2O)_2]^+$	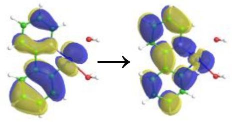
$(Aliz)^{2-}$	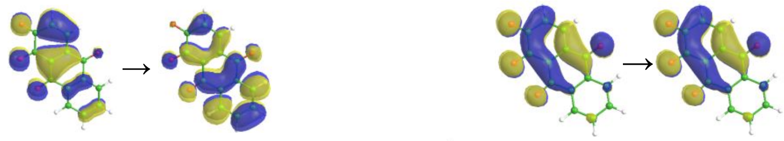
**2** $[(PhPy)Pt(BrCat)]^-$	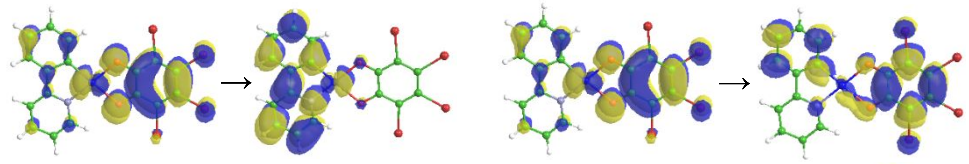
$[(PhPy)Pt(H_2O)(BrCat)]^-$	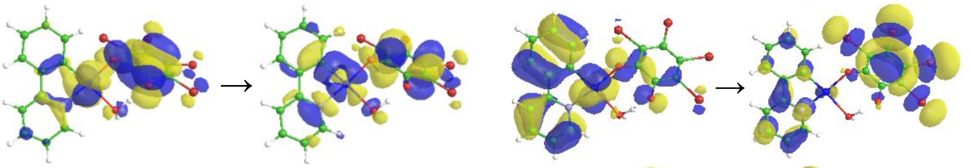
$(BrCat)^{2-}$	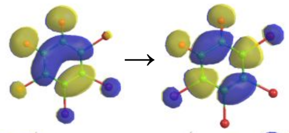
**3** $[(NH_3)_2Pt(Aliz)]$	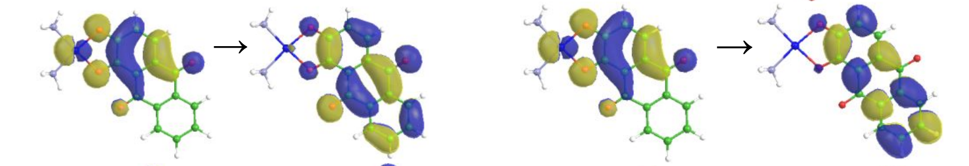
$[(NH_3)_2Pt(H_2O)(Aliz)]^-$	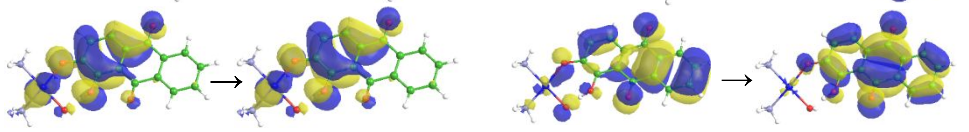

Table S1: Wavelength, λ (nm), oscillator strengths, *f*, and main transitions computed in water and DMSO

	Band	λ_{water}	f_{water}	λ_{DMSO}	f_{DMSO}	MO contribution
**1**$_{cis}$ $[(PhPy)Pt(Aliz)]^-$	I	570	0.420	573	0.432	H→L, 99%
	II	445	0.061	459	0.083	H→L+1, 97%
	III	388	0.019	389	0.019	H-1→L+1, 81%
	IV	307	0.166	308	0.167	H-3→L+1,55%; H-1→L+2, 20%
		302	0.153	302	0.171	H-3→L+1, 55%; H-1→L+2, 20%
$[(PhPy)Pt(H_2O)(Aliz)]^-$	I	513	0.311	516	0.322	H→L, 98%
	II	350	0.044	350	0.074	H→L+2, 44%; H-5→L, 31%
		348	0.047	348	0.017	H→L+2 44%; H-7→L, 26%
		317	0.046	316	0.062	H-1→L+2, 74%
		316	0.076	315	0.077	H-9→L, 77%
		313	0.061	313	0.052	H-4→L+1, 40%; H-1→L+3 27%
**1**$_{trans}$ $[(PhPy)Pt(Aliz)]^-$	I	572	0.413	575	0.427	H→L, 98%
		446	0.049	448	0.049	H→L+1, 94%
	II	311	0.024	311	0.020	H-9→L, 85%;
		308	0.287	308	0.300	H-3→L+1, 42%; H-1→L+2, 39%
		293	0.108	294	0.117	H→L+4, 52%; H-5→L+1, 22%
$[(PhPy)Pt(H_2O)(Aliz)]^-$	I	548	0.240	551	0.251	H→L, 98%
	II	377	0.059	378	0.062	H→L, 77%;
		344	0.040	345	0.041	H-3→L+1, 59%; H-2→L+1, 26%
	III	321	0.052	321	0.052	H-4→L+1, 58%
		318	0.045	318	0.052	H→L+4, 64%
		313	0.048	313	0.049	H-1→L+2, 30%; H-2→L+2, 28%
		310	0.040	311	0.040	H-1→L+3, 69%
$[(PhPy)Pt(H_2O)_2]^+$	I	370	0.029	370	0.031	H→L, 96%
	II	302	0.071	302	0.082	H-2→L, 55%; H→L+1, 36%
		295	0.057	295	0.058	H→L+1, 36%; H-3→L, 30%; H-2→L, 28%
		280	0.217	280	0.231	H-3→L 53%; H→L+1 22%
$(Aliz)^{2-}$	I	567	0.327	571	0.341	H→L, 100%;
	II	279	0.495	278	0.515	H-2→L+1, 63%
		272	0.152	272	0.143	H-7→L, 49%
**2** $[(PhPy)Pt(BrCat)]^-$	I	446	0.158	448	0.164	H→L+1, 98%
	II	310	0.106	311	0.113	H-3→L+1, 53%;
		293	0.175	294	0.190	H→L+4, 56%;
$[(PhPy)Pt(H_2O)(BrCat)]^-$	I	323	0.144	323	0.152	H→L+3, 56%
	II	284	0.134	285	0.203	H-7→L, 49%
		282	0.143	283	0.082	H-7→L+1, 27%; H-7→L, 18%
$(BrCat)^{2-}$	I	282	0.138	283	0.147	H→L+3, 85%
		276	0.135	277	0.144	H→L+4, 82%
**3** $[(NH_3)_2Pt(Aliz)]$	I	532	0.270	534	0.278	H→L, 99%
	II	316	0.050	317	0.058	H-7→L, 72%
	III	278	0.186	278	0.198	H-8→L, 34%; H-1→L+1, 39%
		274	0.523	275	0.536	H→L+4, 48%; H-1→L+1, 42%
$[(NH_3)_2Pt(H_2O)(Aliz)]^-$	I	516	0.246	517	0.256	H→L, 97%
	II	340	0.052	340	0.055	H→L+1, 46%; H→L+2, 33%
	III	264	0.529	264	0.535	H-2→L+1, 57%
$[(NH_3)_2Pt(H_2O)]^{2+}$	I	193	0.195	193	0.205	H→L+3, 98%
	II	168	0.104	167	0.108	H-4→L, 88%
		157	0.190	157	0.196	H-5→L, 88%

Figure S5: UV-Vis spectra of different concentration of CT-DNA in PBS buffer solution in the presence of complexes **1-3** (50 μM)

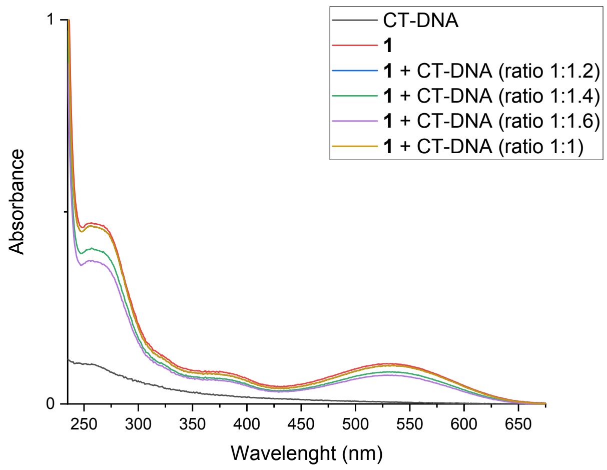

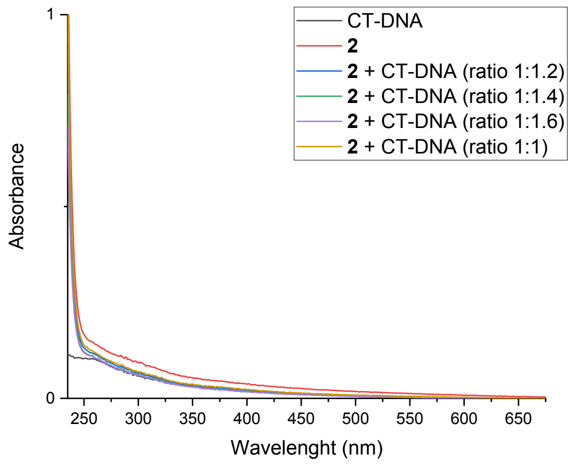

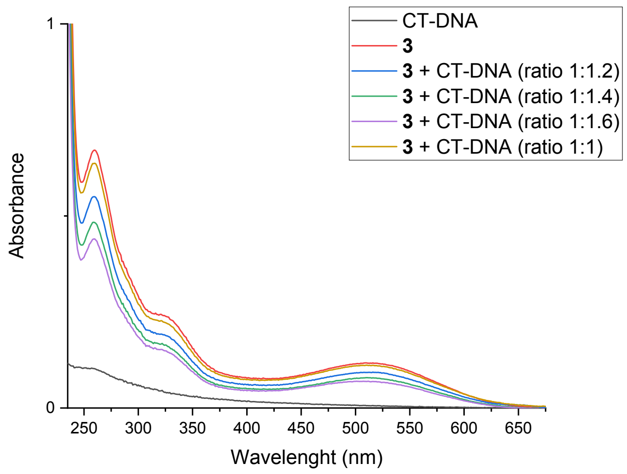

Figure S6: Emission spectra of CT-DNA (10 μM) with EtBr (5 μM) after the addition of increased amount of complexes **1-3**.

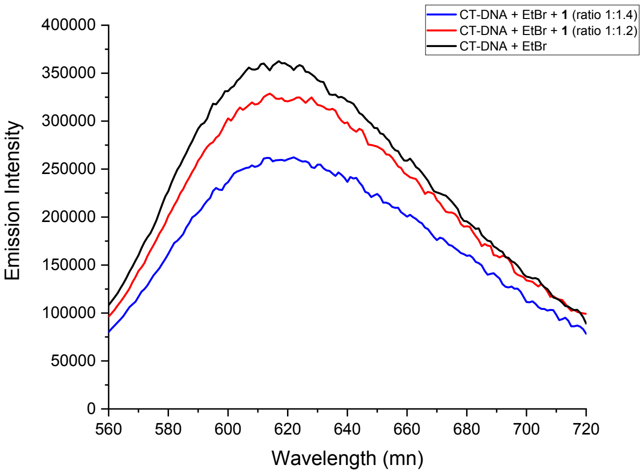

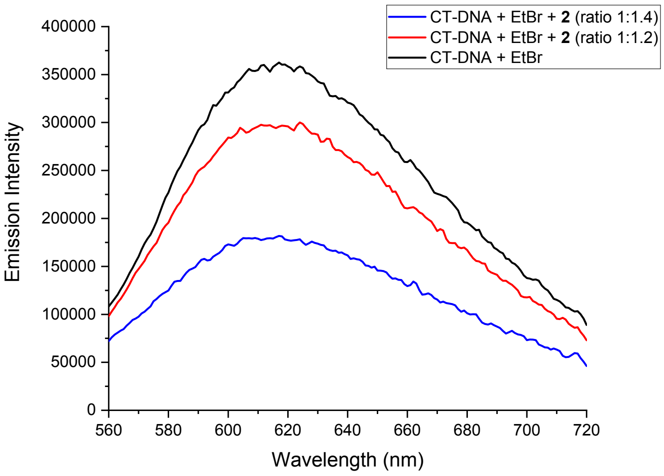

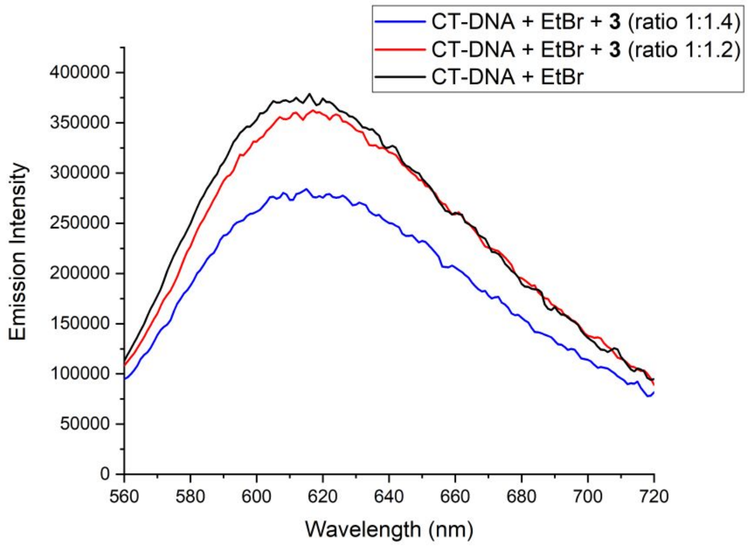
CT-DNA + EtBr + 3 (ratio 1:1.4)
CT-DNA + EtBr + 3 (ratio 1:1.2)
CT-DNA + EtBr
Emission Intensity
400000
350000
300000
250000
200000
150000
100000
50000
0
560
580
600
620
640
660
680
700
720
Wavelength (nm)
